# Immune Architecture of Early Human Aortic Atherosclerosis Reveals Prominent T-Cell and Myeloid Compartments: Beyond Coronaries

**DOI:** 10.1016/j.jacbts.2026.101735

**Published:** 2026-09-22

**Authors:** Tipack Ayothyapattanam Shanmugam, Annemarie Witz, Simang Champramary, Elias Stahl, Nora Göbel, Hauke Horstmann, Bartosz Rylski, Dennis Wolf, Michael Torzewski

**Affiliations:** a Dr. Margarete Fischer-Bosch Institute of Clinical Pharmacology, Stuttgart, Germany; b Faculty of Science, University of Tübingen, Tübingen, Germany; c Institute of Biomedicine, University of Turku, Turku, Finland; d Department of Cardiovascular Surgery, Robert Bosch Hospital, Stuttgart, Germany; e Department of Cardiology and Angiology I, Medical Center, University of Freiburg, Freiburg, Germany; f Department of Laboratory Medicine and Hospital Hygiene, Robert Bosch Hospital, Stuttgart, Germany

**Keywords:** early atherosclerosis, flow cytometry, human aorta, multiplex immunofluorescence, T cells

Early human atherosclerotic lesions represent a critical window in which lipoprotein retention and immune activation begin to shape lesion trajectory. Yet, the leukocyte architecture of these earliest lesions remains poorly characterized because human tissue is scarce and vessel-wall dissociation is technically challenging. Most contemporary human immune atlases^1^ have focused on advanced plaques, frequently using single-cell sequencing approaches, whereas anatomically resolved studies of histologically defined early lesions remain limited. Here, we characterize for the first time the immune landscape of early human aortic lesions (American Heart Association^2^ [AHA] type I-II) by integrating anatomically restricted tissue processing, CD45^+^ enrichment, stringent flow-cytometric quality control, unsupervised clustering, and complementary spatial validation.

Thoracic aortic tissue was obtained from 8 patients undergoing surgery for acute type A aortic dissection (ATAAD) between June 2025 and February 2026 with informed consent and Ethics Committee approval (Medical Faculty, University of Tübingen; ID: 448/2009BO2). Consistent with consensus statements of ATAAD as predominantly a media-centered degenerative process,^3^ these surgical specimens provide rare access to histologically identifiable early intimal lesions. Methodology is outlined in Figure 1A. AHA staging was confirmed by Sudan Black B lipid staining with matched hematoxylin and eosin sections (Figure 1B). To enrich the lesion microenvironment and minimize dilution by uninvolved vessel wall, processing was restricted to lesion-containing intima and the immediately apposed intima–media interface.

**Figure 1:**
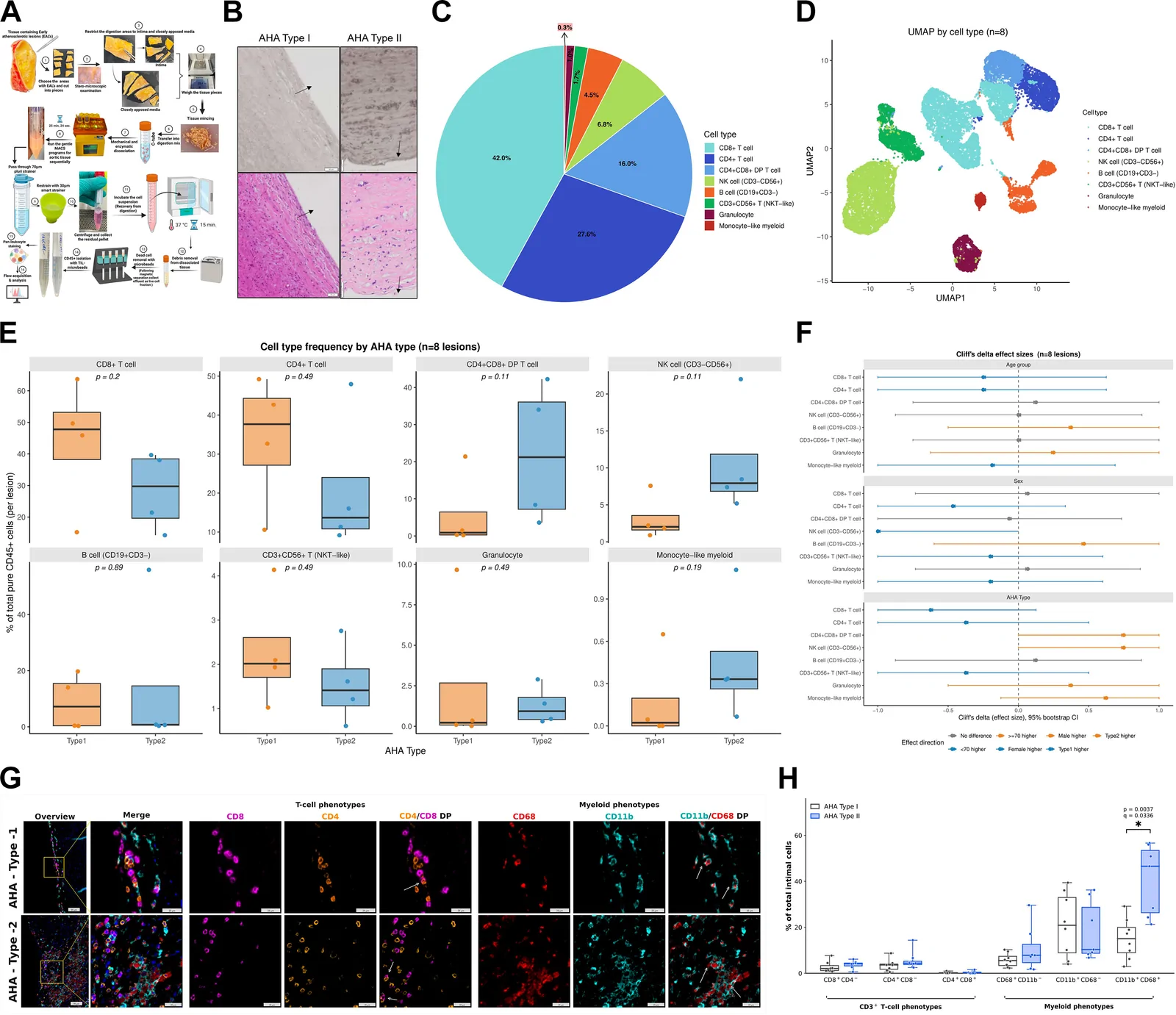
Integrated Flow-Cytometric and Spatial Profiling Reveals Complementary T-Cell and Myeloid Compartments in Early Human Aortic Atherosclerosis (A) Workflow for anatomically restricted lesion isolation and immune profiling. (B) Representative Sudan Black B (top) and paired hematoxylin and eosin (bottom) defining lesion stage (scale bars: 50 μm); arrows indicate lumen. (C) Composition of 140,651 recovered CD45^+^ cells from 8 lesions. (D) UMAP of annotated immune populations. (E) Per-lesion frequencies (Wilcoxon rank-sum test) in AHA (type I, n = 4; type II, n = 4) lesions. (F) Cliff's delta by age, sex, and AHA type; whiskers indicate bootstrap 95% CIs (5,000 resamples). (G) Representative multiplex immunofluorescence of intimal CD4^+^, CD8^+^, CD4^+^CD8^+^ DP, CD68^+^, CD11b^+^, and CD11b^+^CD68^+^ DP phenotypes in type I (upper) and type II (lower) lesions; overview/magnified fields, 95/35 μm. (H) Intimal phenotype quantification (T cells: type I, n = 7; type II, n = 8; myeloid: type I, n = 8; type II, n = 7). AHA = American Heart Association; CD = cluster of differentiation; DP = double positive; UMAP = Uniform Manifold Approximation and Projection.

After dissociation, debris/dead-cell depletion, and CD45^+^ enrichment, cells were stained with a focused leukocyte lineage panel (CD45, CD3, CD4, CD8, CD19, CD56, CD11b, CD15) and acquired on a BD FACSLyric according to established immunologic quality control principles.^4^

Flow cytometry data were analyzed in R using flowCore,^5^ flowAI, flowVS, and FlowSOM. Cluster identities were assigned from canonical marker expression and verified by backgating.^6^ Samples underwent independent quality control before computational integration. The final analytical cohort comprised 8 lesions and 140,651 viable CD45^+^ singlets, with recovery and composition assessed at the individual-lesion level.

The recovered leukocyte compartment was strongly enriched for T cells. Integrated analysis identified CD8^+^ T cells (42.0%), CD4^+^ T cells (27.6%), and CD4^+^CD8^+^ double-positive (DP) T cells (16.0%) as the major populations, together representing 85.6% of the final CD45^+^ population; NK cells, B cells, CD3^+^CD56^+^ NKT-like cells, granulocytes, and monocyte-like myeloid cells comprised smaller fractions (Figures 1C and 1D). Particularly notable was the detection of a CD4^+^CD8^+^ DP T-cell compartment. Stringent lineage gating and comparative backgating supported a distinct FlowSOM-defined DP phenotype within the CD3^+^ T-cell compartment, which was independently corroborated by spatial CD4/CD8 colocalization (Figure 1G).

At the individual lesion level, none of the 8 populations differed significantly between AHA type I-II lesions (Figure 1E), emphasizing substantial heterogeneity within these early stages. Cliff's delta analyses further explored effect magnitude according to age, sex, and AHA stage (Figure 1F). B-cell and granulocyte frequencies tended to be higher in older individuals, whereas several T-cell and myeloid populations showed heterogeneous directional effects across metadata strata. Because confidence intervals were broad and frequently crossed zero, these analyses should be considered hypothesis-generating rather than evidence of definitive subgroup differences. Yet, these observations warrant validation in larger cohorts.

Multiplex immunofluorescence complemented the dissociation-based analysis by confirming intimal CD4^+^, CD8^+^, and CD4^+^CD8^+^ phenotypes and revealing abundant CD68^+^ and CD11b^+^ myeloid cells despite their low cytometric recovery (Figures 1G and 1H). This discrepancy likely reflects differential loss of tissue-associated, lipid-laden myeloid cells during processing, as cytometry measures recovered viable leukocytes rather than absolute tissue abundance. Spatial quantification (Visiopharm v2026.02.0) showed significantly increased CD11b^+^CD68^+^ DP cells in AHA type II lesions (*P* = 0.0037; FDR-adjusted q = 0.0336), whereas T-cell phenotypes showed no significant stage-associated differences (Figure 1H), demonstrating complementary adaptive and myeloid dimensions of early plaque immunity.

In conclusion, these findings indicate that immune organization is already established during the earliest histologically recognizable stages of human atherogenesis. Instead of portraying nascent lesions as simply T-cell or macrophage dominant, our integrated data reveal coexisting but differently captured immune compartments: prominent CD8^+^, CD4^+^, and unexpectedly abundant CD4^+^CD8^+^ DP T-cell populations within the recovered leukocyte fraction, alongside an established intimal myeloid compartment that becomes more prominent with progression to type II lesions. The limited cohort and ATAAD surgical context constrain generalizability; nevertheless, the convergence of flow cytometric and spatial evidence identifies early human atherosclerosis as an immunologically active and heterogeneous state. Most importantly, it highlights the CD4^+^CD8^+^ DP T-cell phenotype and evolving myeloid accumulation as priorities for future mechanistic investigation. Our work establishes the foundation for such investigation and supports prioritizing early atherosclerotic lesions for in-depth immune interrogation at the crossroads of atherogenic lipid retention and vessel wall remodeling.
